# On-Treatment Grade 3–4 Neutropenia and Clinical Outcomes with Trifluridine/Tipiracil in Refractory Metastatic Colorectal Cancer: ReTrITA Real-World Evidence

**DOI:** 10.32604/or.2026.080964

**Published:** 2026-08-13

**Authors:** Carlo Signorelli, Michele Basso, Annunziato Anghelone, Maria Alessandra Calegari, Alessandro Passardi, Chiara Gallio, Alessandro Bittoni, Jessica Lucchetti, Lorenzo Angotti, Emanuela Di Giacomo, Ina Valeria Zurlo, Cristina Morelli, Emanuela Dell’Aquila, Adele Artemi, Donatello Gemma, Domenico Cristiano Corsi, Alessandra Emiliani, Marta Ribelli, Federica Mazzuca, Giulia Arrivi, Federica Zoratto, Maria Grazia Morandi, Fiorenza Santamaria, Manuela Dettori, Antonella Cosimati, Rosa Saltarelli, Alessandro Minelli, Emanuela Lucci-Cordisco, Mario Giovanni Chilelli

**Affiliations:** 1 Medical Oncology Unit, S. Rosa Hospital, ASL Viterbo, Viterbo, Italy; 2 Oncologia Medica, Comprehensive Cancer Center, Fondazione Policlinico Universitario Agostino Gemelli-IRCCS, Rome, Italy; 3 Department of Medical Oncology, IRCCS Istituto Romagnolo per lo Studio dei Tumori (IRST) “Dino Amadori”, Meldola, Italy; 4 Division of Medical Oncology, Policlinico Universitario Campus Bio-Medico, Rome, Italy; 5 Medical Oncology, “Vito Fazzi” Hospital, Lecce, Italy; 6 Medical Oncology Unit, Department of Systems Medicine, Tor Vergata University Hospital, Rome, Italy; 7 IRCCS Regina Elena National Cancer Institute, Rome, Italy; 8 Medical Oncology Unit, ASL Frosinone, Sora, Italy; 9 Medical Oncology, Isola Tiberina Hospital, Gemelli Isola, Rome, Italy; 10 Department of Clinical and Molecular Medicine, Oncology Unit, Sant’ Andrea University Hospital, Sapienza University of Rome, Rome, Italy; 11 UOC Oncologia, Ospedale Santa Maria Goretti, ASL Latina, Latina, Italy; 12 Medical Oncology Unit, San Camillo de Lellis Hospital, ASL Rieti, Rieti, Italy; 13 UOC Oncology A, Policlinico Umberto I, Rome, Italy; 14 Experimental Medicine, Network Oncology and Precision Medicine, Department of Experimental Medicine, Sapienza University of Rome, Rome, Italy; 15 Medical Oncology Department, Ospedale Oncologico Armando Businco, Cagliari, Italy; 16 Medical Oncology Department, UO Oncologia Universitaria della Casa della Salute di Aprilia, Aprilia, Italy; 17 UOC Oncology, San Giovanni Evangelista Hospital, ASL RM5, Tivoli, Italy; 18 Medical Oncology Dept., UO Oncologia, Ospedale San Paolo, ASL RM4, Civitavecchia, Italy; 19 UOC Genetica Medica, Dipartimento di Scienze della Vita e Sanità Pubblica, Fondazione Policlinico Universitario Agostino Gemelli, IRCCS, Rome, Italy

**Keywords:** Trifluridine/tipiracil, metastatic colorectal cancer, neutropenia, real-world evidence, survival outcomes, prognostic biomarkers

## Abstract

**Background**: Trifluridine/tipiracil (T) is a standard treatment for refractory metastatic colorectal cancer (mCRC). In randomized trials, treatment-emergent neutropenia has been associated with improved outcomes, suggesting a potential link with drug activity. However, evidence from routine clinical practice remains limited. This sub-analysis of the multicenter ReTrITA study evaluated the association between severe neutropenia and clinical outcomes in a real-world setting. **Methods**: Patients with refractory mCRC treated with T within the ReTrITA cohort were included. Patients were stratified according to the occurrence of grade 3–4 neutropenia. Overall survival (OS) and progression-free survival (PFS) were estimated using the Kaplan–Meier method and compared with the log-rank test. Hazard ratios (HRs) and 95% confidence intervals (CIs) were calculated using Cox models. **Results**: Among 843 patients, 271 (32.1%) developed grade 3–4 neutropenia. The occurrence of severe neutropenia was significantly associated with improved survival outcomes. Median OS was 10.9 months versus 7.6 months, HR 0.64; 95% CI, 0.55–0.75; *p* < 0.0001), and median PFS was 4.3 months versus 3.3 months (HR 0.63; 95% CI, 0.55–0.73; *p* < 0.0001) in patients with versus without neutropenia, respectively. **Conclusions**: In this large real-world cohort, grade 3–4 neutropenia during T treatment was associated with significantly improved OS and PFS, supporting its role as a potential on-treatment prognostic marker. Prospective studies are warranted to confirm these findings.

## 1 Introduction

Colorectal cancer (CRC) continues to represent a major contributor to the global cancer burden, remaining one of the leading causes of cancer-related morbidity and mortality worldwide. Even though screening methods, early diagnosis, and treatment options have all gotten a lot better, CRC still causes a lot of cancer deaths, especially in the advanced stages [1]. According to the recent GLOBOCAN estimates, there are more than 1.9 million new cases of CRC and about 930,000 deaths from CRC each year around the world. This makes CRC the third most common cancer diagnosis and the second most common cause of cancer-related death [1]. Projections show that the disease burden will grow even more, with incidence and mortality rates expected to rise by 63–73% by 2040 due to an ageing population and changes in lifestyle-related risk factors [1].

In the United States, CRC remains the third most common type of cancer and the second most common cause of death from cancer. Current estimates show that there are about 150,000 to 155,000 new cases and more than 52,000 deaths each year, even though the number of cases and deaths among older adults is going down overall [2]. Of concern, epidemiological analyses have consistently reported a progressive increase in CRC incidence among individuals younger than 50 years, with an average annual rise of approximately 1–2% over the past two decades, underscoring that CRC can no longer be considered exclusively a disease of the elderly [3,4]. This epidemiological shift highlights that CRC can no longer be considered a disease confined to older populations.

A similar burden is observed across Europe, where CRC remains among the most frequently diagnosed cancers, with approximately 520,000 new cases and more than 245,000 deaths each year [1,3]. Despite the implementation of population-based screening strategies and improvements in early diagnosis, a substantial proportion of patients still present with advanced disease or eventually develop metastatic spread.

Indeed, despite advancements in screening, diagnosis, and multimodal treatment approaches, approximately 50–60% of patients with CRC ultimately develop metastatic disease, and many will encounter disease progression following standard first- and second-line systemic therapies. In the refractory metastatic colorectal cancer (mCRC) setting, therapeutic options remain limited, and treatment decisions must carefully balance expected clinical benefit, toxicity profile, and preservation of quality of life [4,5].

In the context of metastatic disease, therapeutic advancements over the last twenty years—including cytotoxic chemotherapy, targeted therapies, and immunotherapy—have markedly prolonged survival. However, the majority of patients eventually experience disease progression after standard first- and second-line treatments. For patients with refractory mCRC, the choice of treatment in later lines is influenced by previous therapies, molecular characteristics, clinical status, and expected toxicity, with the primary objective of sustaining survival while ensuring quality of life [6,7,8].

Trifluridine/tipiracil (T) is an orally administered antineoplastic agent approved for the treatment of patients with refractory mCRC [9]. Its clinical efficacy was established in the pivotal phase III RECOURSE trial, which randomized 800 heavily pretreated patients in a 2:1 ratio to receive T or placebo. This study showed that T significantly improved overall survival compared to placebo, with a median overall survival of 7.1 months versus 5.3 months (hazard ratio [HR] 0.68; 95% CI, 0.58–0.81; *p* < 0.001). It also showed a statistically significant improvement in progression-free survival [10]. The Asian phase II J003 trial later confirmed these results, showing that patients treated with T had a 21% lower risk of death than those who received a placebo. This supports the idea that the survival benefit can be seen in different groups of people [11,12].

Trifluridine/tipiracil has demonstrated a consistent survival benefit in patients with refractory mCRC and is currently recommended by international guidelines as a standard treatment option in later lines of therapy, both as monotherapy and, more recently, the result adds to recent data, notably those from the SUNLIGHT trial [8], which showed better efficacy when T was administered in combination with bevacizumab, showing an ongoing development of post-progression therapy regimens in mCRC [13,14,15,16,17,18,19].

In randomised clinical trials, T has demonstrated a tolerable safety profile, with haematologic toxicity—especially neutropenia—being the most commonly reported adverse event [20,21]. In the RECOURSE trial, grade 3–4 neutropenia manifested in approximately 35–40% of patients, whereas febrile neutropenia was infrequent [10]. Significantly, post hoc analyses of the RECOURSE and SUNLIGHT studies indicated that the emergence of treatment-related neutropenia correlated with enhanced clinical outcomes, including extended overall survival and progression-free survival [20,21,22,23,24,25]. These observations prompted the hypothesis that neutropenia may signify sufficient drug exposure and serve as a potential prognostic and pharmacodynamic indicator of treatment efficacy, rather than merely constituting an adverse event.

Most of the evidence supporting neutropenia’s role as a potential prognostic factor comes from randomised controlled trials, which may not fully reflect the diversity of patients treated in regular clinical practice. People in the real world often include older patients, people with multiple health problems, and patients whose bone marrow reserve has been damaged by a lot of previous treatments. These are all things that could affect both toxicity patterns and survival outcomes.

Within this context, the ReTrITA project (Regorafenib and Trifluridine/Tipiracil in Italian Patients) represents a nationwide multicenter real-world initiative including 1156 patients with refractory mCRC treated with regorafenib and/or T across 17 Italian oncology centers. The present analysis focuses on a predefined sub-cohort of 843 patients treated with T monotherapy, providing a unique opportunity to explore, in a large and unselected real-world population, the association between grade 3–4 neutropenia and survival outcomes [26,27].

This sub-analysis was therefore designed to evaluate whether the prognostic impact of severe neutropenia observed in randomized clinical trials can be confirmed in routine clinical practice, and to further clarify its role as a potential prognostic and pharmacodynamic on-treatment marker in patients with refractory metastatic colorectal cancer treated with T.

## 2 Patients and Methods

### 2.1 Study Overview

This study is based on the ReTrITA dataset, a nationwide multicenter retrospective cohort including patients with refractory mCRC treated in routine clinical practice across 17 Italian oncology centers between 2012 and 2023.

**Figure 1:**
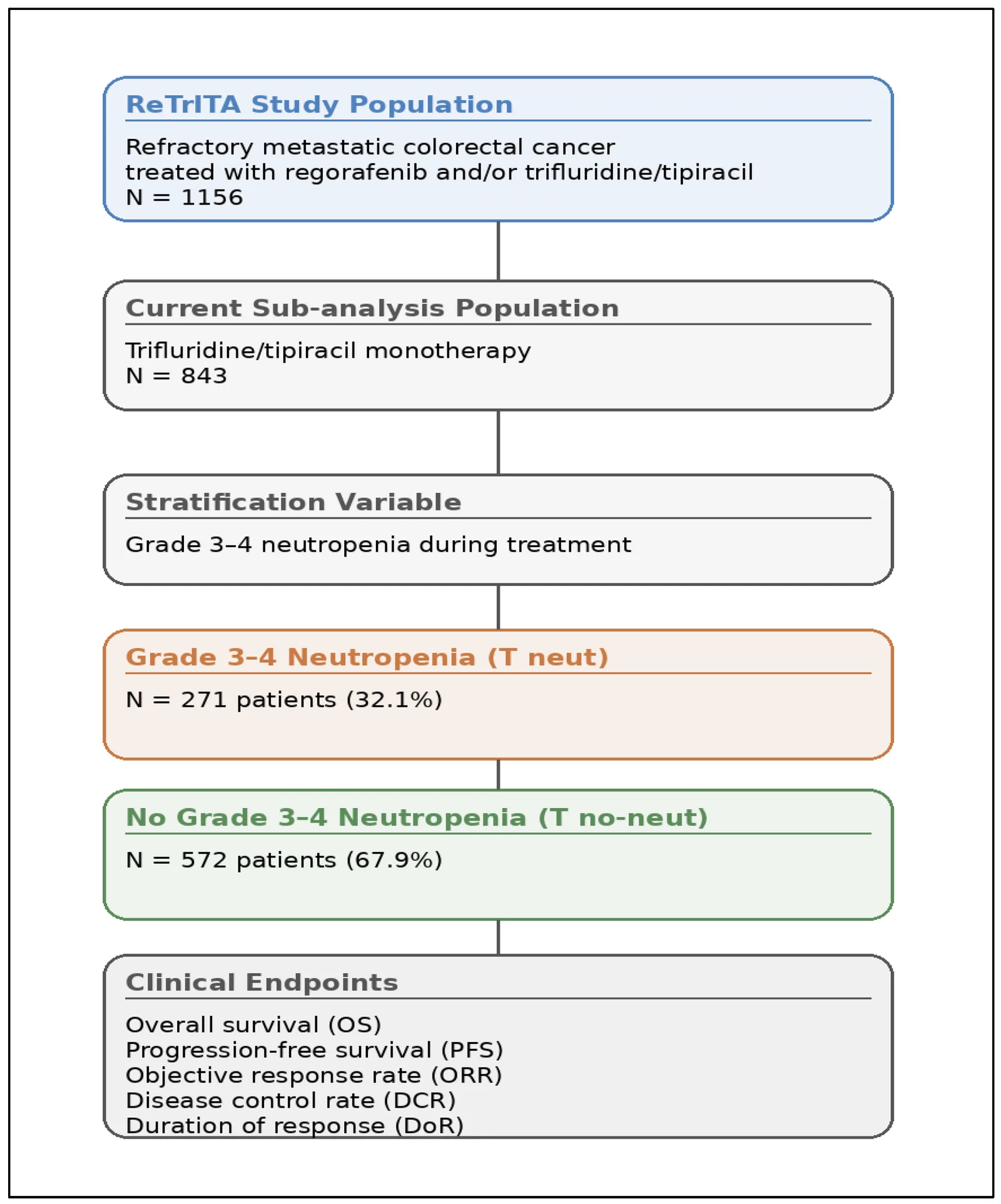
Study design of the ReTrITA sub-analysis. Among 1156 patients included in the overall ReTrITA cohort, 843 patients treated with trifluridine/tipiracil monotherapy were eligible for the present analysis and were stratified according to the occurrence of grade 3–4 neutropenia into T neut (n = 271) and T no-neut (n = 572) groups. Abbreviations: T, trifluridine/tipiracil; N, number.

The present analysis represents a predefined sub-analysis of the ReTrITA cohort and focuses exclusively on patients treated with T as monotherapy in the refractory setting. Eligible patients were adults (≥18 years) with histologically confirmed metastatic colorectal adenocarcinoma who had progressed after, or were intolerant to, standard therapies, including fluoropyrimidines, oxaliplatin, irinotecan, anti-VEGF (Vascular Endothelial Growth Factor) agents, and anti-EGFR (Epidermal Growth Factor Receptor) therapies when indicated. Patients who received T in combination with other systemic agents or within clinical trials were excluded from this sub-analysis. The study design is illustrated in Fig. 1.

Patients were retrospectively stratified according to the occurrence of grade 3–4 neutropenia during treatment with T, as defined by the National Cancer Institute Common Terminology Criteria for Adverse Events (NCI CTCAE), version 4.0. Baseline demographic, clinical, pathological, and treatment-related variables were collected from medical records at each participating center.

### 2.2 Outcome Measures

The primary endpoints of this sub-analysis were overall survival (OS) and progression-free survival (PFS). Overall survival was defined as the time from the start of T treatment to death from any cause. Progression-free survival was defined as the interval from treatment initiation to the first occurrence of radiologically and/or clinically documented disease progression or death, whichever occurred first.

Secondary endpoints included objective response rate (ORR), disease control rate (DCR), and duration of response (DoR). The ORR was defined as the proportion of patients achieving a complete response (CR) or partial response (PR), while DCR included patients with CR, PR, or stable disease (SD), according to Response Evaluation Criteria in Solid Tumors (RECIST), version 1.1, when radiological assessments were available in routine clinical practice.

Duration of response (DoR) was defined as the time from the first documented objective response (CR or PR) to disease progression or death from any cause. Tumor assessments were performed according to local clinical practice and imaging schedules, reflecting the real-world nature of the study.

The association between severe neutropenia and survival and efficacy outcomes was subsequently evaluated.

### 2.3 Treatment Administration

Trifluridine/tipiracil was administered orally according to standard clinical practice and approved dosing schedules. The recommended dose was 35 mg/m^2^ twice daily on days 1–5 and 8–12 of each 28-day cycle [9]. Dose reductions, treatment delays, or temporary interruptions were allowed at the discretion of the treating physician based on hematologic and non-hematologic toxicities.

Treatment was continued until disease progression, unacceptable toxicity, deterioration of performance status, or patient decision to discontinue therapy [9]. Hematologic parameters, including neutrophil counts, were monitored according to institutional practice, typically prior to each treatment cycle and as clinically indicated.

### 2.4 Statistical Analysis

Descriptive statistics were used to summarize baseline characteristics and treatment-related variables. Continuous variables were reported as medians with ranges, while categorical variables were expressed as frequencies and percentages.

Overall survival and PFS were estimated using the Kaplan–Meier method, and compared using the log-rank test. Hazard ratios (HRs) and corresponding 95% confidence intervals (CIs) were calculated using Cox proportional hazards models to evaluate the association between the occurrence of grade 3–4 neutropenia and survival outcomes.

Group comparisons (patients with vs. without grade 3–4 neutropenia) were performed using the chi-square test or Fisher’s exact test for categorical variables and the Mann–Whitney U test for continuous variables, as appropriate. All statistical tests were two-sided, and a *p*-value ≤ 0.05 was considered statistically significant. Subgroup analyses were exploratory and descriptive in nature, and no formal statistical tests for interaction were performed.

A propensity score matching (PSM) analysis was performed to reduce the impact of baseline confounding between patients with and without grade 3–4 neutropenia. The propensity score was estimated using sex, age ≥ 70 years, ECOG performance status, RAS (rat sarcoma viral oncogene homolog) status, primary tumor location, MSI/MMR status, metastatic disease sites, and front-line antibody exposure. Patients were matched 1:1 using nearest-neighbor matching without replacement, with a caliper width of 0.2 of the standard deviation of the logit of the propensity score.

Statistical analyses were conducted using MedCalc software (version 19.4; MedCalc Software, Ostend, Belgium). Given the retrospective and exploratory nature of the study, no adjustment for multiple comparisons was applied.

Neutropenia was modeled as a time-fixed on-treatment variable (occurrence at any time during treatment), and not as a time-dependent covariate.

### 2.5 Ethical Approval

The ReTrITA study was conducted in accordance with the Declaration of Helsinki and received approval from the Ethics Committee of Area 4 Lazio, Rome, Italy (protocol number 29-2024; approval date: 4 March 2024).

All data were anonymized prior to analysis to ensure patient confidentiality. Due to the retrospective nature of the study, the requirement for written informed consent was waived for patients who were deceased, unreachable, or declined consent, in accordance with applicable data protection regulations.

## 3 Results

### 3.1 Patient Characteristics

A total of 843 patients treated with T were included in this sub-analysis, of whom 271 (32.1%) developed grade 3–4 neutropenia during treatment (T neut group), while 572 (67.9%) did not (T no-neut group). Baseline demographic, clinical, and treatment-related characteristics of the two groups are summarized in Table 1.

**Table 1:** Patient characteristics according to grade 3–4 neutropenia.

Patient Characteristics	T Neut	T No-Neut	*p*-Value
**Total, N (%)**	271 (100)	572 (100)	<0.0001
**Age, years**			
** Median (min–max)**	68 (42–87)	68 (30–88)	>0.999
**Age**			
** ≥70 yrs, N (%)**	135 (49.8)	283 (49.5)	0.9266
** <70 yrs, N (%)**	136 (50.2)	289 (50.5)	
**Sex**			
** Female, N (%)**	120 (44.3)	233 (40.7)	0.3300
** Male, N (%)**	151 (55.7)	339 (59.3)	
**RAS status**			
** Wild type, N (%)**	91 (33.6)	205 (35.8)	0.2422
** Mutant type, N (%)**	174 (64.2)	343 (60.0)	
** Unknown, N (%)**	6 (2.2)	24 (4.2)	
**Primary tumor location**			
** Right side, N (%)**	91 (33.6)	189 (33.0)	0.9845
** Left side, N (%)**	113 (41.7)	239 (41.8)	
** Rectum, N (%)**	67 (24.7)	144 (25.2)	
**MMR**			
** dMMR, N (%)**	7 (2.6)	15 (2.6)	0.4595
** pMMR, N (%)**	170 (62.7)	383 (67.0)	
** Unknown, N (%)**	94 (34.7)	174 (30.4)	
**PS ECOG**			
** 0, N (%)**	68 (25.1)	158 (27.6)	0.3859
** 1, N (%)**	178 (65.7)	324 (56.6)	
** 2, N (%)**	25 (9.2)	90 (15.7)	
**Prior adjuvant therapy**			
** Yes, N (%)**	90 (33.2)	161 (28.1)	0.1334
** No, N (%)**	181 (66.8)	411 (71.9)	
**Metastatic disease sites**			
** Liver only, N (%)**	33 (12.2)	92 (16.1)	0.2548
** Liver + other, N (%)**	134 (49.4)	282 49.3)	
** Others, N (%)**	102 (37.6)	197 (34.4)	
** Unknown, N (%)**	2 (0.7)	1 (0.2)	
**CT 1 line regimen**			
** Monochemotherapy, N (%)**	24 (8.9)	36 (6.3)	0.1695
** Doublet chemotherapy, N (%)**	224 (82.7)	467 (81.6)	
** Triplet chemotherapy, N (%)**	22 (8.1)	60 (10.5)	
** Unknown, N (%)**	1 (0.4)	9 (1.6)	
**CT 2 line regimen**			
** Monochemotherapy, N (%)**	24 (8.9)	58 (10.1)	0.5603
** Doublet chemotherapy, N (%)**	217 (80.1)	434 (75.9)	
** Triplet chemotherapy, N (%)**	4 (1.5)	13 (2.3)	
** Unknown, N (%)**	26 (9.6)	67 (11.7)	
**Rechallenge therapy**			
** yes, N (%)**	22 (8.1)	34 (5.9)	0.2368
** no, N (%)**	249 (91.9)	538 (94.1)	
**Biological agents 1 line**			
** Anti-EGFR use, N (%)**	72 (26.6)	140 (24.5)	0.1507
** Anti-VEGF use, N (%)**	165 (60.9)	326 (57.0)	
** None, N (%)**	33 (12.2)	99 (17.3)	
** Unknown, N (%)**	1 (0.4)	7 (1.2)	
**Biological agents 2 line**			
** Anti-EGFR use, N (%)**	16 (5.9)	36 (6.3)	0.5376
** Anti-VEGF use, N (%)**	174 (64.2)	361 (63.1)	
** None, N (%)**	70 (25.8)	138 (24.1)	
** Unknown, N (%)**	11 (4.1)	37 (6.5)	

Note: Baseline demographic, clinical, tumor-related, and treatment characteristics of patients with refractory metastatic colorectal cancer treated with trifluridine/tipiracil (T), stratified according to the occurrence of grade 3–4 neutropenia. Data are reported as number (percentage) unless otherwise specified. Comparisons between groups were performed using the chi-square test or Fisher’s exact test for categorical variables and the Mann–Whitney U test for continuous variables, as appropriate. Abbreviations: N, number; RAS, (rat sarcoma viral oncogene homolog); dMMR, deficient Mismatch Repair; pMMR, proficient Mismatch Repair; PS, Performance Status; ECOG, Eastern Cooperative Oncology Group; CT, chemotherapy; EGFR, Epidermal Growth Factor Receptor; VEGF, Vascular Endothelial Growth Factor.

The median age was 68 years in both groups, with a comparable age distribution; approximately half of the patients were aged ≥ 70 years (49.8% in the T neut group vs. 49.5% in the T no-neut group; *p* = 0.9266). Sex distribution was also balanced, with males representing 55.7% of the T neut group and 59.3% of the T no-neut group.

RAS mutational status was similar between groups. In the T neut and T no-neut cohorts, 33.6% and 35.8% of patients, respectively, harbored RAS wild-type tumors, while RAS mutations were detected in 64.2% and 60.0% of patients; RAS status was unknown in a small proportion of cases. Primary tumor location was well balanced, with comparable proportions of right-sided, left-sided, and rectal primaries across the two groups.

The mismatch repair (MMR) status was available for the majority of patients. Deficient MMR (dMMR) tumors were uncommon in both cohorts (2.6% in each group), while proficient MMR (pMMR) tumors accounted for 62.7% of the T neut group and 67.0% of the T no-neut group; MMR status was unknown in approximately one-third of patients.

Baseline performance status was similar between groups. Most patients had an ECOG (Eastern Cooperative Oncology Group) performance status of 0–1, accounting for 90.8% of the T neut group and 84.2% of the T no-neut group. Prior adjuvant chemotherapy had been administered in 33.2% of patients in the T neut group and 28.1% in the T no-neut group.

With regard to disease burden, the distribution of metastatic sites did not differ significantly between groups. Liver-only metastases were observed in 12.2% of patients in the T neut group and 16.1% in the T no-neut group, while liver plus extrahepatic involvement was present in approximately half of patients in both cohorts; other metastatic patterns accounted for the remaining cases.

Treatment history before T was comparable between groups. In the first-line setting, the majority of patients had received doublet chemotherapy (82.7% in the T neut group vs. 81.6% in the T no-neut group), with smaller proportions treated with monochemotherapy or triplet regimens. Similar patterns were observed for second-line chemotherapy. Rechallenge strategies had been used in a minority of patients (8.1% vs. 5.9%, respectively).

Exposure to biological agents was well balanced. In the first-line setting, anti-VEGF agents were the most frequently used targeted therapies (60.9% in the T neut group and 57.0% in the T no-neut group), followed by anti-EGFR agents; a minority of patients had not received biological therapy. Comparable distributions were observed in the second-line setting.

Overall, baseline demographic characteristics, tumor features, disease burden, and prior treatment exposures were well balanced between patients who developed grade 3–4 neutropenia and those who did not, supporting the robustness of subsequent comparative analyses of clinical outcomes.

Baseline characteristics before and after PSM are summarized in Table 2. Before matching, moderate imbalances were observed across several variables. After matching, all baseline covariates were well balanced between the two groups, with standardized mean differences below 0.10, indicating an adequate balance. After matching, 270 patients with grade 3–4 neutropenia were compared with 270 patients without neutropenia, achieving adequate balance across baseline covariates (Table 2).

**Table 2:** Baseline characteristics before and after propensity score matching.

Variable	Pre-PSM Neutropenia (N = 271)	Pre-PSM No Neutropenia (N = 572)	SMD	Post-PSM Neutropenia (N = 270)	Post-PSM No Neutropenia (N = 270)	SMD
**Age ≥ 70 years, N (%)**	135 (49.8%)	283 (49.5%)	0.01	134 (49.6%)	135 (50.0%)	0.01
**Sex (female), N (%)**	120 (44.3%)	233 (40.7%)	0.07	119 (44.1%)	118 (43.7%)	0.01
**ECOG PS ≥ 1, N (%)**	203 (74.9%)	414 (72.4%)	0.06	201 (74.4%)	203 (75.2%)	0.02
**RAS mutated, N (%)**	174 (64.2%)	343 (60.0%)	0.09	173 (64.1%)	172 (63.7%)	0.01
**Tumor location**						
Right-sided, N (%)	91 (33.6%)	189 (33.0%)	0.01	90 (33.3%)	91 (33.7%)	0.01
Left-sided, N (%)	113 (41.7%)	239 (41.8%)	0.00	113 (41.9%)	112 (41.5%)	0.01
Rectum, N (%)	67 (24.7%)	144 (25.2%)	0.01	67 (24.8%)	67 (24.8%)	0.00
**MMR status**						
dMMR, N (%)	7 (2.6%)	15 (2.6%)	0.00	7 (2.6%)	6 (2.2%)	0.02
pMMR, N (%)	170 (62.7%)	383 (67.0%)	0.09	169 (62.6%)	170 (63.0%)	0.01
**Metastatic disease sites**						
Liver only, N (%)	33 (12.2%)	92 (16.1%)	0.11	33 (12.2%)	34 (12.6%)	0.01
Liver + other, N (%)	134 (49.4%)	282 (49.3%)	0.00	133 (49.3%)	134 (49.6%)	0.01
Other, N (%)	102 (37.6%)	197 (34.4%)	0.07	102 (37.8%)	101 (37.4%)	0.01
**Front-line biological therapy**						
Anti-VEGF, N (%)	165 (60.9%)	326 (57.0%)	0.08	164 (60.7%)	165 (61.1%)	0.01
Anti-EGFR, N (%)	72 (26.6%)	140 (24.5%)	0.05	71 (26.3%)	72 (26.7%)	0.01
None, N (%)	33 (12.2%)	99 (17.3%)	0.14	33 (12.2%)	33 (12.2%)	0.00

Note: Baseline demographic and clinical characteristics of patients with and without grade 3–4 neutropenia are reported before and after propensity score matching. The propensity score was estimated using sex, age ≥ 70 years, ECOG performance status, RAS status, primary tumor location, MSI/MMR status, metastatic disease sites, and front-line biological therapy exposure. Patients were matched 1:1 using nearest-neighbor matching with a caliper of 0.2. Standardized mean differences were used to assess covariate balance, with values < 0.10 indicating adequate balance between groups. Abbreviations: N, number; PSM, propensity score matching; RAS, rat sarcoma viral oncogene homolog; dMMR, deficient Mismatch Repair; pMMR, proficient Mismatch Repair; PS, Performance Status; ECOG, Eastern Cooperative Oncology Group; EGFR, Epidermal Growth Factor Receptor; VEGF, Vascular Endothelial Growth Factor; SMD, standardized mean differences.

### 3.2 Survival Outcomes

**Figure 2:**
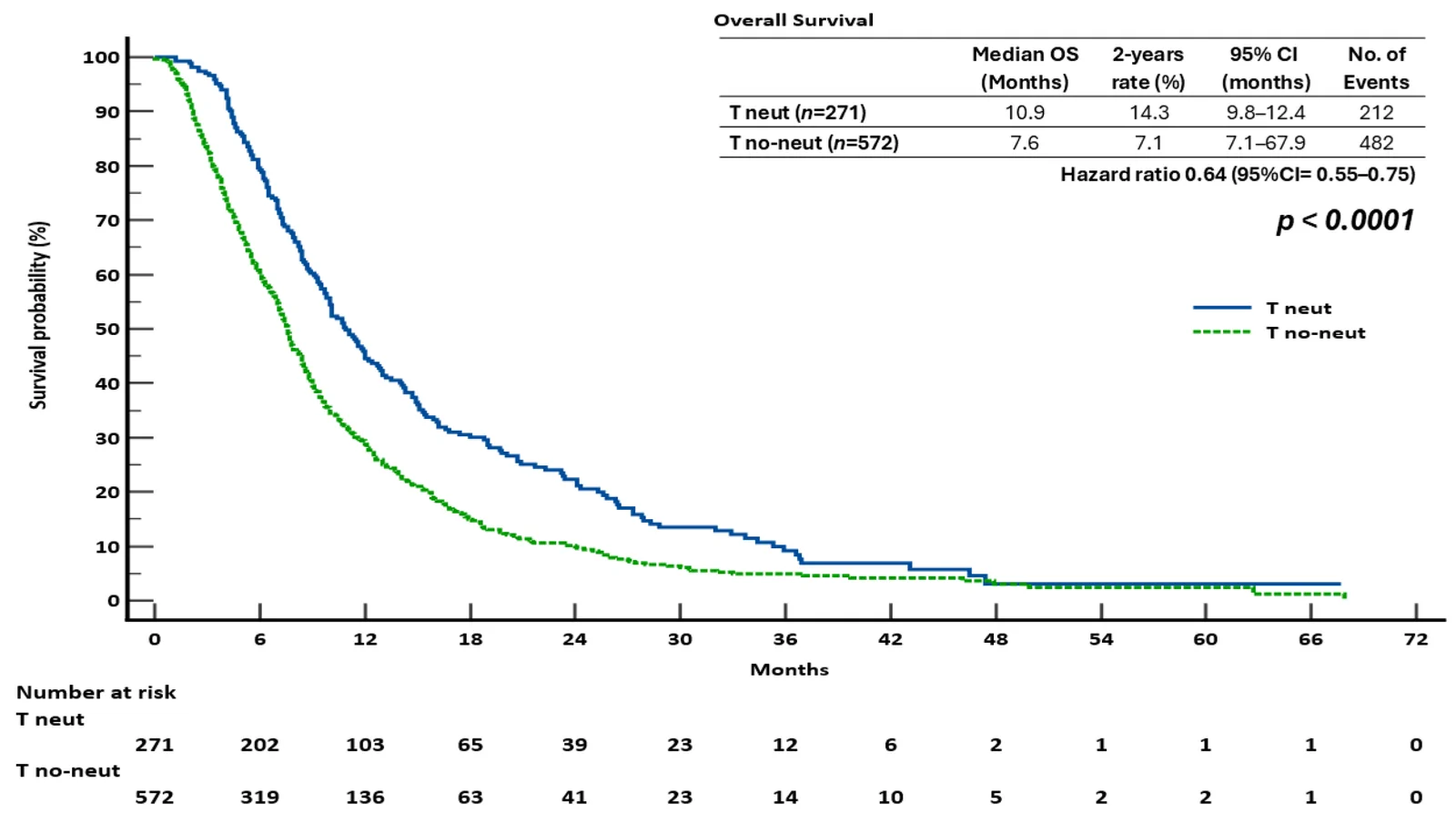
Kaplan–Meier estimates of overall survival in patients with refractory metastatic colorectal cancer treated with trifluridine/tipiracil, stratified according to the occurrence of grade 3–4 neutropenia. Survival curves compare patients who developed grade 3–4 neutropenia (T neut) with those who did not (T no-neut). Differences between groups were assessed using the log-rank test. Abbreviations: OS, overall survival; CI, Confidence Interval; T, Trifluridine/tipiracil; T neut, group of patients who developed grade 3–4 neutropenia; T no-neut, group of patients who did not develop grade 3–4 neutropenia.

At the time of data cutoff, survival outcomes differed significantly between patients who developed grade 3–4 neutropenia during T treatment and those who did not. Overall survival was significantly longer in patients who experienced grade 3–4 neutropenia (T neut group) compared with those without severe neutropenia (T no-neut group). Median OS was 10.9 months (95% CI, 9.8–12.4) in the T neut group versus 7.6 months (95% CI, 7.1–67.9) in the T no-neut group. Kaplan–Meier analysis demonstrated a clear separation of survival curves, with a statistically significant difference between groups (log-rank χ^2^ value = 31.43; DF (degrees of freedom) = 1; *p* < 0.0001) (Fig. 2). In Cox proportional hazards regression analysis, the occurrence of grade 3–4 neutropenia was associated with a 36% reduction in the risk of death, corresponding to an HR of 0.64 (95% CI, 0.55–0.75), confirming severe neutropenia as a strong prognostic factor for improved OS.

**Figure 3:**
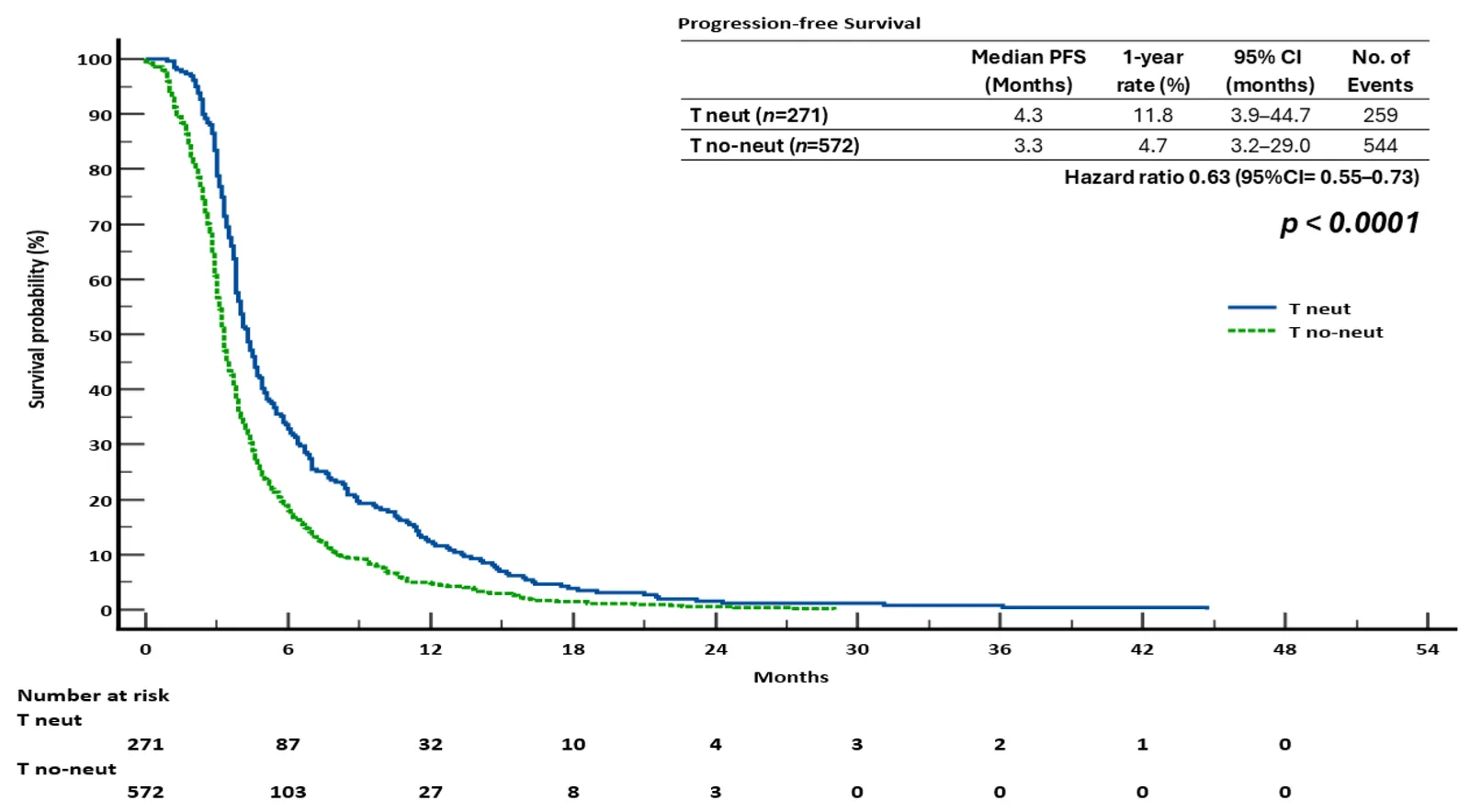
Kaplan–Meier estimates of progression-free survival (PFS) in patients with refractory metastatic colorectal cancer treated with trifluridine/tipiracil (T), stratified according to the occurrence of grade 3–4 neutropenia. Progression-free survival curves compare patients with grade 3–4 neutropenia (T neut) and those without severe neutropenia (T no-neut). Differences between groups were evaluated using the log-rank test. Abbreviations: PFS, progression-free survival; CI, Confidence Interval; T, Trifluridine/tipiracil; T neut, group of patients who developed grade 3–4 neutropenia; T no-neut, group of patients who did not developed grade 3–4 neutropenia.

A similar pattern was observed for PFS. Patients in the T neut group experienced a significantly longer median PFS compared with those in the T no-neut group (4.3 months; 95% CI, 3.9–44.7 vs. 3.3 months; 95% CI, 3.2–29.0, respectively). Kaplan–Meier estimates showed a marked and early divergence of PFS curves (log-rank χ^2^ value = 37.67; DF = 1; *p* < 0.0001) (Fig. 3). Consistently, grade 3–4 neutropenia was associated with a significantly reduced risk of disease progression or death, with a hazard ratio of 0.63 (95% CI, 0.55–0.73) in Cox regression analysis.

### 3.3 Summary of Efficacy

The efficacy outcomes of this sub-analysis are summarised in Table 3. In line with expectations for a refractory mCRC population treated with T, objective tumor responses were infrequent in both treatment groups.

**Table 3:** Clinical outcomes stratified by the occurrence of grade 3–4 neutropenia.

Clinical Outcomes		T Neut	T No-Neut
**OS**	mOS (months)	10.9	7.6
	3y-OS (%)	4.4	2.4
	2y-OS (%)	14.3	7.1
	HR (95% CI)	0.64 (0.55–0.75)	
	*p*-Value	**<0.0001**	
**PFS**	mPFS (months)	4.3	3.3
	1y-PFS (%)	11.8	4.7
	2y-PFS (%)	1.4	0.5
	HR (95% CI)	0.63 (0.55–0.73)	
	*p*-Value	**<0.0001**	
**ORR**	PR + CR (%)	5.9	1.0
	*p*-Value	**0.0164**	
**DCR**	PR + CR + SD (%)	34.7	21.2
	*p*-Value	**0.0021**	
**DoR**	Median DoR (months)	10.5	7.2
	HR (95% CI)	0.84 (0.29–2.38)	
	*p*-Value	0.7432	

Note: Efficacy outcomes in patients with refractory metastatic colorectal cancer treated with trifluridine/tipiracil (T), stratified according to the occurrence of grade 3–4 neutropenia. Outcomes include overall survival (OS), progression-free survival (PFS), objective response rate (ORR), disease control rate (DCR), and duration of response (DoR). Survival outcomes were estimated using the Kaplan–Meier method and compared using the log-rank test. Hazard ratios (HRs) and corresponding 95% confidence intervals (CIs) were derived from Cox proportional hazards regression models. Tumor response was assessed according to RECIST version 1.1, when available. Abbreviations: OS, overall survival; HR, Hazard Ratio; CI, Confidence Interval; PFS, progression-free survival; ORR, objective response rate; PR, partial response; CR, complete response; DCR, disease control rate; SD, stable disease; DoR, duration of response.

The ORR was low overall but significantly higher in patients who developed grade 3–4 neutropenia compared with those who did not (5.9% vs. 1.0%; *p* = 0.0164), while remaining consistent with the limited tumor shrinkage activity of T in late-line settings. The DCR, on the other hand, was significantly higher in patients with grade 3–4 neutropenia (34.7% vs. 21.2%; *p* = 0.0021). In contrast, no significant difference was observed in DoR between the two groups (10.5 vs. 7.2 months; *p* = 0.7432). This suggests that patients who developed grade 3–4 neutropenia during treatment had a more lasting antitumor effect.

However, the DCR was much higher and the DoR was much longer for patients with grade 3–4 neutropenia. The higher DCR observed in patients with grade 3–4 neutropenia did not translate into a significantly longer DoR. This discrepancy may be explained by the limited number of patients achieving an objective response and the consequent reduced statistical power for DoR analysis. This is consistent with the big improvements in OS and PFS that were seen in this group. These results show that the survival benefit of severe neutropenia is not due to more tumour shrinkage, but rather to longer disease control and response durability.

### 3.4 Comparative Analysis of Subgroups Survival

To further investigate the consistency of the correlation between grade 3–4 neutropenia and survival outcomes, subgroup analyses were conducted for both OS and PFS. Patients receiving T were categorised based on clinically significant baseline characteristics, and survival outcomes were compared between the T neut and T no-neut groups within each subgroup.

**Figure 4:**
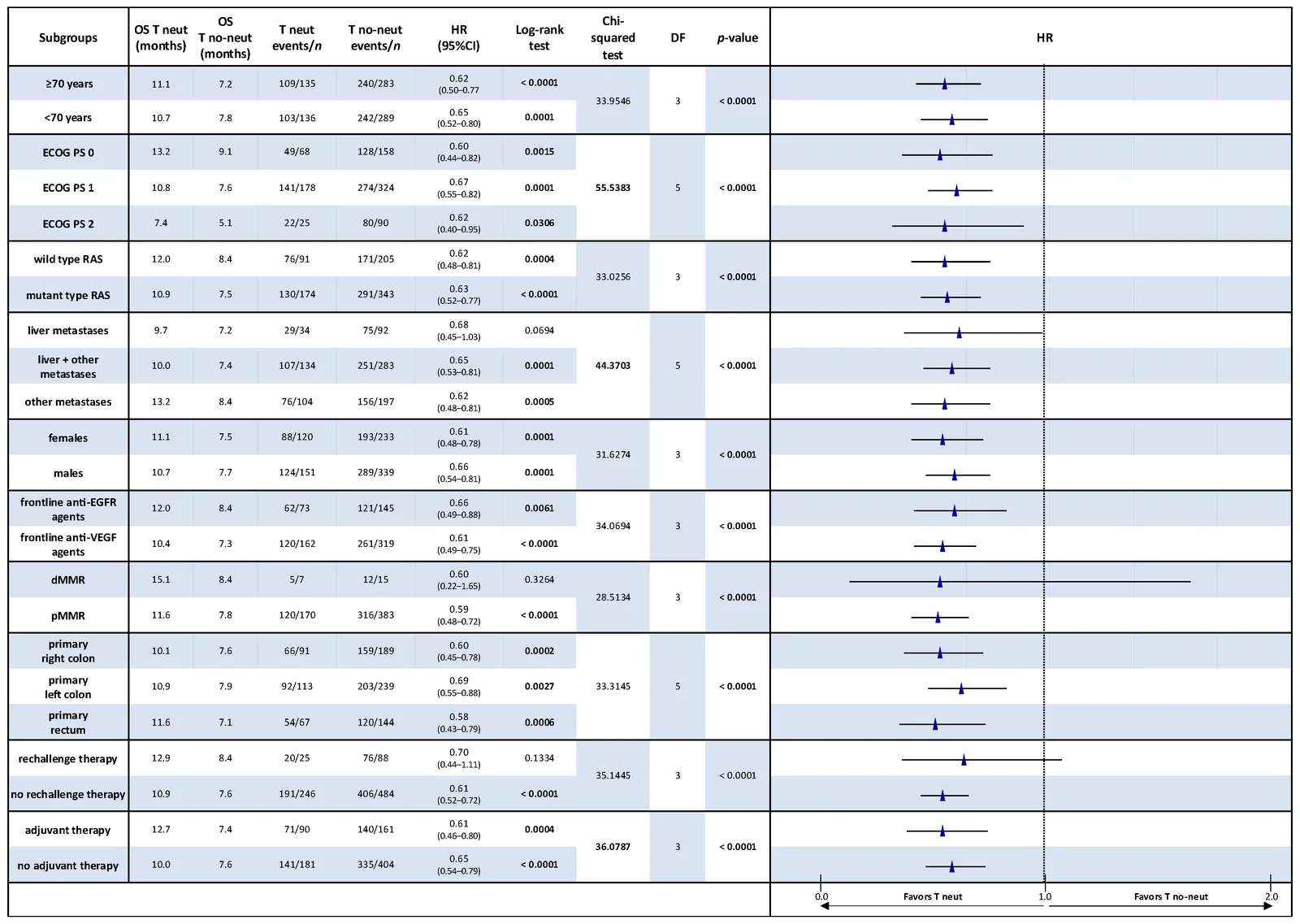
Overall survival by grade 3–4 neutropenia status across clinical subgroups. Forest plot of overall survival (OS) according to the occurrence of grade 3–4 neutropenia in patients with refractory metastatic colorectal cancer treated with trifluridine/tipiracil (T). Hazard ratios (HRs) and corresponding 95% confidence intervals (CIs) are shown for the comparison between patients who developed grade 3–4 neutropenia (T neut) and those who did not (T no-neut) across predefined clinical subgroups. HRs < 1 indicate a survival benefit in favor of the T neut group. Abbreviations: n, number; OS, overall survival; DF, degrees of freedom; RAS, rat sarcoma viral oncogene homolog; dMMR, deficient Mismatch Repair; pMMR, proficient Mismatch Repair; PS, Performance Status; ECOG, Eastern Cooperative Oncology Group; EGFR, Epidermal Growth Factor Receptor; VEGF, Vascular Endothelial Growth Factor.

Across the majority of predefined subgroups, the occurrence of grade 3–4 neutropenia was consistently associated with a favorable survival outcome. For OS, the effect consistently favoured patients with severe neutropenia, as indicated by HRs predominantly below unity across most subgroups. This benefit was noted regardless of age, sex, ECOG performance status, primary tumour location, and RAS mutational status, indicating that the prognostic significance of neutropenia was predominantly independent of initial demographic and tumor-related variables (Fig. 4).

**Figure 5:**
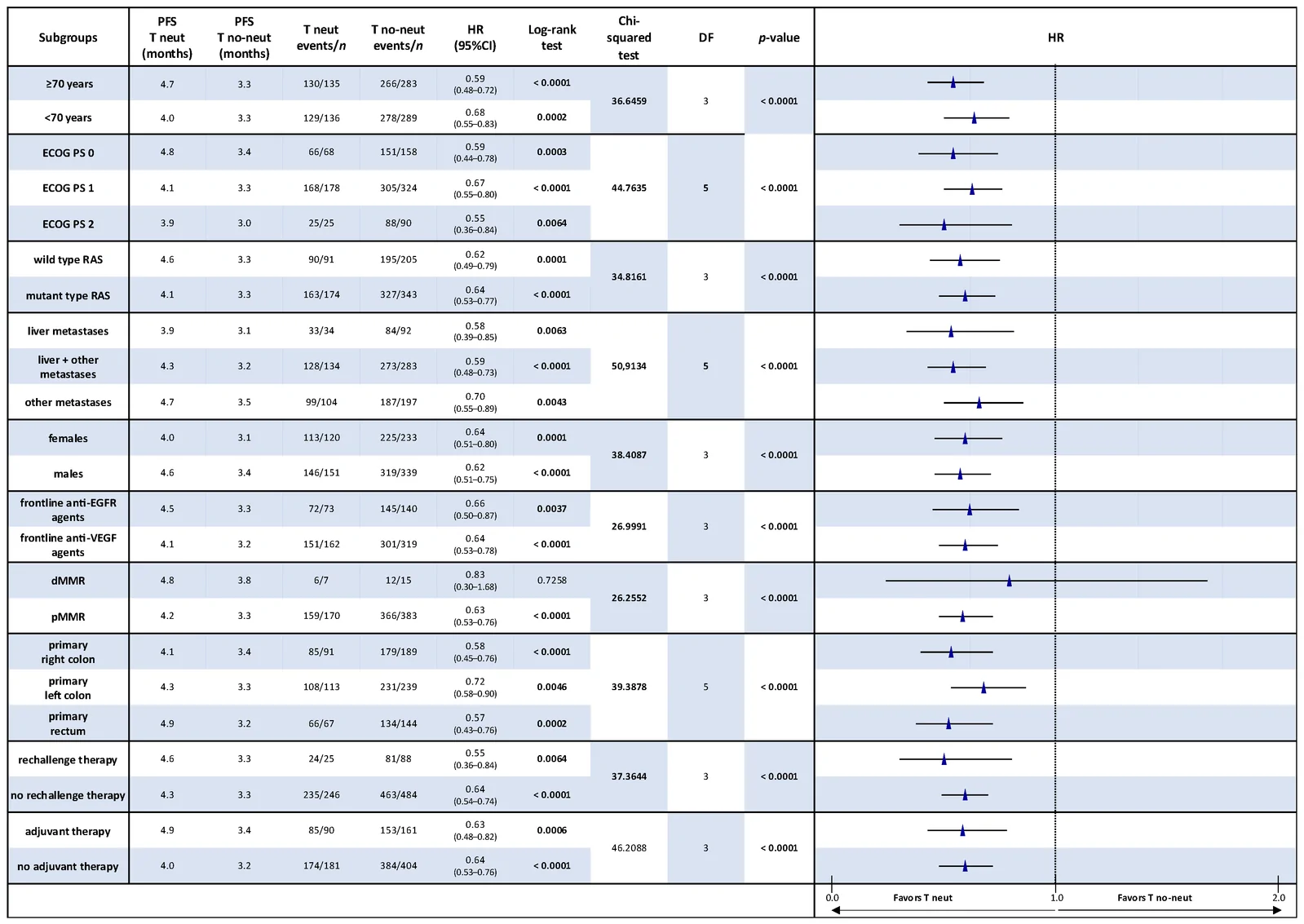
Progression-free survival by grade 3–4 neutropenia status across clinical subgroups. Forest plot of progression-free survival (PFS) according to the occurrence of grade 3–4 neutropenia in patients with refractory metastatic colorectal cancer treated with trifluridine/tipiracil (T). Hazard ratios (HRs) and corresponding 95% confidence intervals (CIs) are presented for each predefined subgroup, comparing patients with grade 3–4 neutropenia (T neut) versus those without severe neutropenia (T no-neut). HRs < 1 indicate a reduced risk of disease progression or death associated with the development of grade 3–4 neutropenia. Abbreviations: n, number; PFS, progression-free survival; DF, degrees of freedom; RAS, rat sarcoma viral oncogene homolog; dMMR, deficient Mismatch Repair; pMMR, proficient Mismatch Repair; PS, Performance Status; ECOG, Eastern Cooperative Oncology Group; EGFR, Epidermal Growth Factor Receptor; VEGF, Vascular Endothelial Growth Factor.

A comparable trend was noted for PFS. Subgroup analyses showed that patients with grade 3–4 neutropenia had a lower risk of disease progression or death, and this effect was similar across all clinically relevant subgroups in terms of size and direction. It is important to note that the association between neutropenia and better PFS held true no matter what treatment the patient had received before or how many metastases they had, which supports the strength of this finding in a real-world setting (Fig. 5).

Importantly, no qualitative heterogeneity was observed between grade 3–4 neutropenia and the main baseline clinical variables was observed. For all predefined subgroups, HR estimates for both OS and PFS consistently favoured patients who developed severe neutropenia, with no subgroup demonstrating a statistically significant reversal of the effect. There was some variation in effect size, probably because of differences in the size of the subgroup samples and the baseline risk. However, the overall consistency of the direction and size of the HRs supports the strength and generalisability of the correlation between grade 3–4 neutropenia and higher survival outcomes.

**Figure 6:**
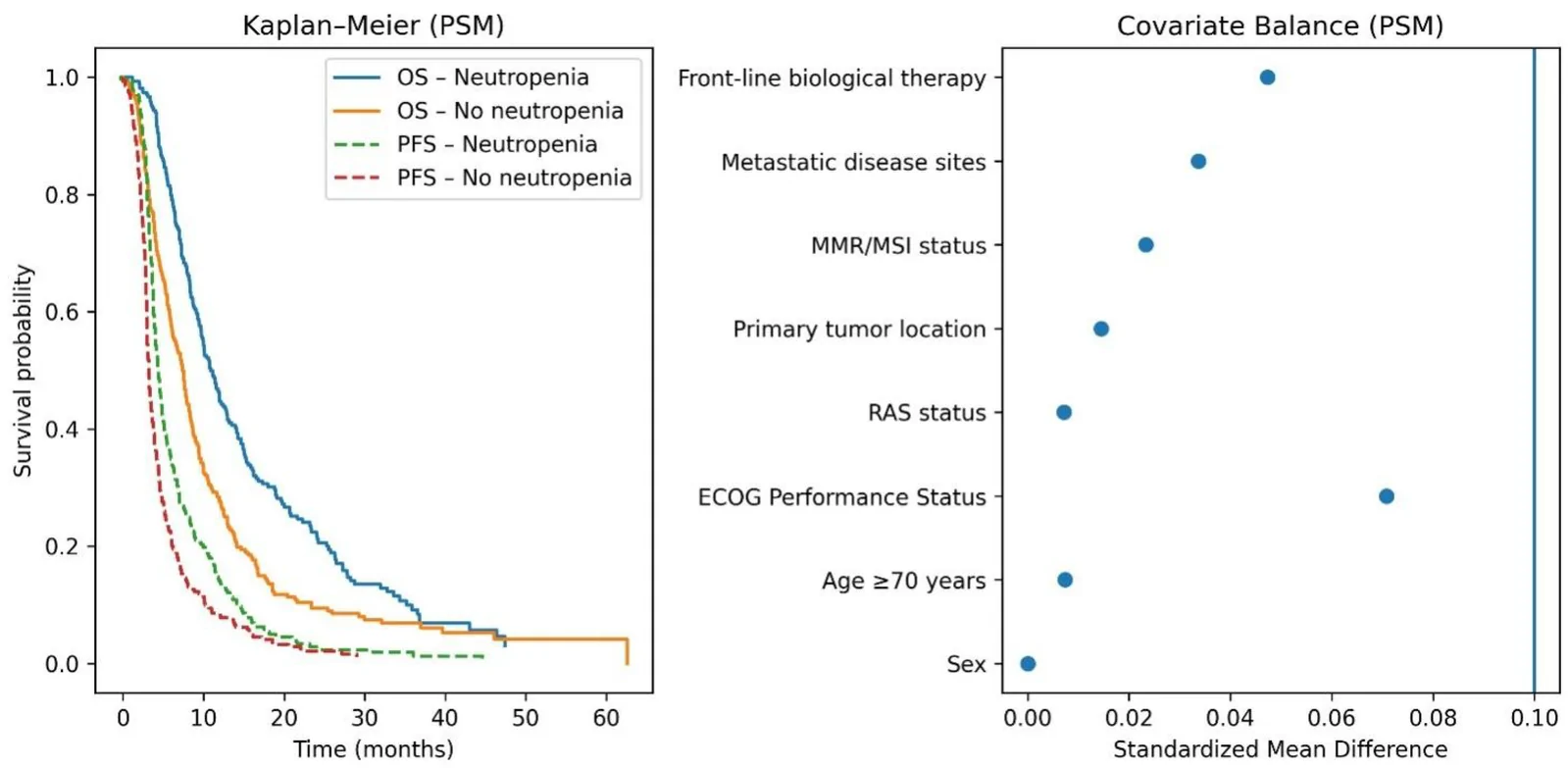
Survival outcomes and covariate balance after propensity score matching. (**Left**): Kaplan–Meier curves for overall survival (OS, solid lines) and progression-free survival (PFS, dashed lines) in patients with and without grade 3–4 neutropenia after propensity score matching (PSM). (**Right**): standardized mean differences (SMD) for baseline covariates included in the propensity score model. The vertical reference line indicates the threshold of 0.10, below which covariates are considered adequately balanced. Abbreviations: RAS, rat sarcoma viral oncogene homolog; MMR, Mismatch Repair; MSI, Microsatellite Instability; ECOG, Eastern Cooperative Oncology Group.

To confirm the robustness of these findings, a PSM analysis was performed. Kaplan–Meier curves for OS and PFS in the matched cohort, along with covariate balance assessed by standardized mean differences, are shown in Fig. 6.

In the matched cohort, the occurrence of grade 3–4 neutropenia remained significantly associated with improved survival outcomes. Median OS was 10.9 months in the neutropenia group versus 7.6 months in the non-neutropenia group (HR 0.64, 95% CI 0.55–0.75; *p* < 0.0001). Similarly, median PFS was 4.3 months versus 3.3 months, respectively (HR 0.63, 95% CI 0.55–0.73; *p* < 0.0001).

## 4 Discussion

### 4.1 Key Findings: What Did We Demonstrate with This ReTrITA Sub-Analysis?

This large multicenter real-world sub-analysis demonstrates that the occurrence of grade 3–4 neutropenia during T therapy is associated with improved clinical outcomes in patients with refractory mCRC. These findings were robust across survival endpoints and supported by highly significant log-rank tests and clinically meaningful HRs, indicating a substantial prognostic impact of severe neutropenia in this real-world cohort.

Patients who developed severe neutropenia experienced significantly longer OS and PFS, together with higher ORR and DCR and prolonged DoR.

These findings are clinically relevant, suggesting that the observed survival advantage is not driven by increased tumor shrinkage, but rather by more sustained disease control—consistent with the known activity profile of T in late-line settings. Subgroup analyses revealed that the positive correlation between severe neutropenia and survival outcomes was consistent across clinically significant strata, with no indication of a qualitative heterogeneity, thereby affirming the robustness and generalisability of this on-treatment marker.

Importantly, the PSM analysis further strengthened the robustness of our findings. As illustrated in Fig. 6, the survival advantage associated with grade 3–4 neutropenia persisted after adjustment for clinically relevant baseline covariates, and adequate balance between groups was confirmed by standardized mean differences below the predefined threshold. These results suggest that the observed association is unlikely to be solely driven by baseline imbalances.

### 4.2 How Do These Findings Compare with Prior Evidence from Trials and Real-World Studies without Bevacizumab?

The hypothesis that neutropenia may reflect treatment activity rather than pure toxicity emerged from randomized evidence. In the pivotal phase III RECOURSE trial, T enhanced OS compared to placebo in heavily pretreated mCRC, with neutropenia being one of the most prevalent grade ≥ 3 adverse events. Subsequent analyses indicated better outcomes for patients who developed neutropenia. The J003 study also showed similar signals, which supports the idea that treatment-emergent neutropenia could be a useful predictor of outcomes in different groups of people [10,11].

In the real-world setting, several retrospective cohorts evaluating T monotherapy have also reported an association between neutropenia and improved OS and/or PFS, although with heterogeneity in study design, neutropenia definitions (any-grade vs. grade ≥ 3), timing (early-cycle vs. anytime on treatment), and adjustment for confounders [20,28,29,30]. Many reports suggest that neutropenia may function as a pragmatic pharmacodynamic surrogate for adequate exposure, but sample sizes have often been limited and endpoints inconsistently reported, especially for DCR and DoR.

Within this landscape, the present analysis adds weight in three ways: (i) it leverages a large multicenter cohort representative of routine practice; (ii) it demonstrates a convergent signal across OS, PFS, DCR and DoR; and (iii) it supports consistency across subgroups, reducing the likelihood that the association is confined to a narrow clinical phenotype.

As summarized in Table 4, evidence from randomized trials and real-world studies consistently suggests an association between treatment-emergent neutropenia and improved clinical outcomes during T monotherapy. Within this heterogeneous literature, the present ReTrITA sub-analysis represents the largest real-world dataset to date and uniquely demonstrates a convergent benefit across OS, PFS, DCR, and DoR, with consistent findings across clinically relevant subgroups.

**Table 4:** Comparative overview of key studies assessing neutropenia during trifluridine/tipiracil monotherapy (without bevacizumab).

Study	Study Design	Population (N)	Treatment Setting	Definition of Neutropenia	Main Survival Findings (OS/PFS)	Additional Efficacy Signals	Reference
RECOURSE	Phase III randomized controlled trial	800	Refractory mCRC (≥3rd line)	Grade ≥ 3 neutropenia (CTCAE)	OS benefit with T vs. placebo; post hoc analyses showed longer OS and PFS in patients developing neutropenia	ORR low; disease control main contributor	Mayer et al., 2015 [10]
J003	Randomized, double-blind, placebo-controlled	172	Refractory mCRC	Any-grade and grade ≥ 3 neutropenia	Survival benefit with T; exploratory analyses supported association between neutropenia and improved outcomes	ORR uncommon; benefit driven by disease stabilization	Yoshino et al., 2012 [11]
Hamauchi study	Retrospective real-world analysis	~150	Refractory mCRC	Early-onset neutropenia (any-grade/grade ≥ 3)	OS significantly longer in patients developing neutropenia; favorable trend for PFS	No clear ORR increase	Hamauchi et al., 2017 [20]
Italian RWE cohort	Retrospective observational study	<200	Refractory mCRC	Grade ≥ 3 neutropenia	OS benefit observed in patients with severe neutropenia	Limited reporting on DCR/DoR	Giuliani & Bonetti, 2019 [28]
Japanese RWE cohort	Retrospective single-center	~200	Late-line mCRC	Decreased neutrophil percentage/grade ≥ 3	Improved efficacy associated with neutropenia	DCR favored neutropenic patients	Makihara et al., 2019 [29]
Nordic RWE cohort	Retrospective multicenter	<200	Refractory mCRC	Grade ≥ 3 neutropenia	OS benefit suggested; PFS trend favorable	Limited response data	Wallander et al., 2020 [30]
UK RWE cohort	Retrospective multicenter	~300	Refractory mCRC	Grade ≥ 3 neutropenia	OS benefit suggested; heterogeneous PFS results	Exploratory prognostic analyses	Stavraka et al., 2021 [31]
European RWE cohort	Retrospective analysis	<300	Refractory mCRC	Neutropenia during treatment	Neutropenia associated with improved outcomes	Signal on disease control	Domínguez Senín et al., 2023 [32]
ReTrITA sub-analysis (present study)	Multicenter real-world study	843	Refractory mCRC	Grade 3–4 neutropenia (anytime on treatment)	Significant improvement in OS and PFS in T neut vs. T no-neut	Higher DCR and longer DoR; ORR unchanged	Present study

Abbreviations: T, trifluridine/tipiracil; mCRC, metastatic colorectal cancer; OS, overall survival; PFS, progression-free survival; ORR, objective response rate; CTCAE, Common Terminology Criteria for Adverse Events; DCR, disease control rate; DoR, duration of response.

### 4.3 Biological and Pharmacodynamic Interpretation: Why Might Neutropenia Track Benefit?

A pharmacodynamic explanation is biologically plausible. Trifluridine/tipiracil exerts antitumor activity primarily through DNA (deoxyribonucleic acid) incorporation, and systemic exposure to its active component is linked to both efficacy and myelosuppression [31]. Consequently, severe neutropenia may represent an on-treatment indicator of adequate exposure and target engagement, rather than a purely detrimental adverse event. However, in the absence of a comparator arm, a predictive role cannot be established. Similarly, without pharmacokinetic or exposure–response data, the pharmacodynamic interpretation remains hypothetical. The present findings should therefore be interpreted as supporting an association consistent with an on-treatment prognostic marker, rather than establishing causality or predictive value.

An alternative and equally plausible explanation is that treatment-emergent neutropenia reflects cumulative drug exposure and treatment duration rather than a direct biological marker of tumor sensitivity. Patients who continue therapy long enough to develop myelosuppression are, by definition, exposed to a greater amount of treatment, which may itself contribute to improved outcomes. Therefore, the observed association between neutropenia and survival should be interpreted with caution, as the current study design does not allow definitive discrimination between these mechanisms. In contrast, absence of neutropenia could reflect lower effective exposure due to interpatient pharmacokinetic variability, differences in metabolism, altered absorption, conservative dose modifications, or reduced treatment intensity.

This framework also helps reconcile the typical dissociation between response and survival seen with T in late-line mCRC: tumor shrinkage is uncommon, whereas disease stabilization and delayed progression can still translate into survival improvements. In our study, the association of grade 3–4 neutropenia with higher DCR and longer DoR, together with OS/PFS benefit, is consistent with a model in which neutropenia tracks durability of disease control rather than radiographic response.

### 4.4 Clinical Implications in Real-World Practice

These results have practical consequences. First, if clinically manageable, the development of grade 3–4 neutropenia during T treatment should not automatically lead to stopping treatment early. Instead, severe neutropenia may signal patients who are getting better and need careful support to keep them exposed when it is safe.

Second, it is important to keep an eye on cytopenias and manage them according to guidelines in order to avoid losing dose intensity that could have been avoided. Patient safety is still the most important thing, but cutting back on doses too early or stopping them altogether—especially if the patient doesn’t have febrile neutropenia or other serious complications—could make it harder to control the disease long-term.

Third, neutropenia could be added to a dynamic, on-treatment risk stratification approach that works with baseline prognostic factors to help people make decisions about whether to continue treatment, how closely to monitor them, and what kind of supportive care they need.

### 4.5 Strengths and Limitations

The main strengths of this study lie in the large, multicenter real-world cohort and its nationwide design, which allowed us to capture a broad and representative patient population. In addition, we were able to comprehensively evaluate several clinically meaningful efficacy endpoints, including OS, PFS, DCR, and DoR. Notably, the consistency of the observed associations across different patient subgroups further reinforces the robustness and potential generalizability of our findings.

An important aspect of the analysis is the use of PSM, which helped to reduce baseline imbalances between groups and improve the overall internal validity. The fact that the survival advantage associated with neutropenia persisted even after matching strengthens the credibility of this association.

At the same time, several limitations should be considered. As a retrospective study, the analysis is inherently subject to selection bias and residual confounding, which cannot be completely ruled out despite statistical adjustment. Moreover, some clinically relevant variables—such as baseline laboratory parameters and time from metastatic diagnosis—were not consistently available and therefore could not be included in the adjusted models.

Another important limitation is that neutropenia was not modeled as a time-dependent covariate. Since it is a treatment-emergent event, it may be influenced by time-related biases, including immortal time bias and guarantee-time bias, which cannot be fully addressed in this setting. In addition, the lack of standardized timing for laboratory assessments and the variability inherent to real-world data collection may have introduced further heterogeneity and the possibility of misclassification.

We also lacked detailed and consistent information on treatment exposure, such as relative dose intensity, dose modifications, and the use of growth factors. As a result, it was not possible to clearly distinguish whether neutropenia reflects a true pharmacodynamic effect or simply serves as a surrogate marker of treatment exposure and duration.

Despite these limitations, the overall consistency of the results across multiple endpoints, together with the similar direction of effect observed in subgroup analyses and the agreement with previously published randomized and real-world data, support the validity and clinical relevance of our findings.

### 4.6 Future Perspectives

Further validation is necessary to determine if severe neutropenia is solely prognostic or if it can function as a clinically relevant pharmacodynamic biomarker. Future research should include time-dependent analyses, look into early-cycle neutropenia as a predictor, and look into exposure–response relationships. In addition, future studies should investigate the impact of dose modifications and supportive interventions on outcomes, to better clarify whether the observed association reflects pharmacodynamic sensitivity or treatment exposure.

Future analyses should incorporate time-dependent Cox models to more accurately account for the dynamic nature of treatment-emergent neutropenia. Also, adding neutropenia to practical algorithms may help find the right level of treatment and get the best control of the disease in mCRC that doesn’t respond to treatment.

## 5 Conclusions

In this large multicenter real-world sub-analysis of the ReTrITA study, the development of grade 3–4 neutropenia during T therapy was consistently associated with improved clinical outcomes in patients with refractory mCRC. Patients who experienced severe neutropenia achieved significantly longer OS and PFS, together with higher DCR and longer DoR, while ORR remained low and comparable between groups.

Importantly, the survival benefit associated with neutropenia was observed across clinically relevant subgroups, supporting its robustness and generalizability in routine clinical practice. The consistency of these findings after PSM supports the role of neutropenia as a potential on-treatment prognostic and pharmacodynamic marker.

In conclusion, grade 3–4 neutropenia during T therapy may function as a potential prognostic and pharmacodynamic on-treatment marker of treatment efficacy rather than merely representing treatment-related toxicity.

Taken together, the present results reinforce the clinical relevance of careful hematologic monitoring during T treatment and support a more nuanced interpretation of severe neutropenia in late-line mCRC, potentially informing treatment optimization and future prospective validation studies.

## Data Availability

The data to support the results reported in this study are available from the corresponding author on reasonable request.

## Abbreviations

CI	confidence interval
CRC	colorectal cancer
CR	complete response
CT	chemotherapy
CTCAE	Common Terminology Criteria for Adverse Events
dMMR	deficient mismatch repair
DCR	disease control rate
DF	degrees of freedom
DNA	deoxyribonucleic acid
DoR	duration of response
ECOG	Eastern Cooperative Oncology Group
EGFR	Epidermal Growth Factor Receptor
HR	Hazard ratio
mCRC	metastatic colorectal cancer
MMR	mismatch repair
MSI	Microsatellite Instability
N	number
ORR	objective response rate
OS	overall survival
PFS	progression-free survival
pMMR	proficient mismatch repair
PR	partial response
PS	Performance Status
PSM	Propensity Score Matching
RAS	rat sarcoma viral oncogene homolog
RECIST	Response Evaluation Criteria in Solid Tumors
ReTrITA	Regorafenib and Trifluridine/Tipiracil in Italian Patients
SD	stable disease
SMD	standardized mean differences
T	Trifluridine/tipiracil
T neut	group of patients who developed grade 3–4 neutropenia
T no-neut	group of patients who did not developed grade 3–4 neutropenia
VEGF	Vascular Endothelial Growth Factor
yrs	years
